# PKA Compartmentalization via AKAP220 and AKAP12 Contributes to Endothelial Barrier Regulation

**DOI:** 10.1371/journal.pone.0106733

**Published:** 2014-09-04

**Authors:** Mariya Y. Radeva, Daniela Kugelmann, Volker Spindler, Jens Waschke

**Affiliations:** Institute of Anatomy and Cell Biology, Ludwig-Maximilians-University Munich, Munich, Germany; University of Illinois at Chicago, United States of America

## Abstract

cAMP-mediated PKA signaling is the main known pathway involved in maintenance of the endothelial barrier. Tight regulation of PKA function can be achieved by discrete compartmentalization of the enzyme via physical interaction with A-kinase anchoring proteins (AKAPs). Here, we investigated the role of AKAPs 220 and 12 in endothelial barrier regulation. Analysis of human and mouse microvascular endothelial cells as well as isolated rat mesenteric microvessels was performed using TAT-Ahx-AKAPis peptide, designed to competitively inhibit PKA-AKAP interaction. *In vivo* microvessel hydraulic conductivity and *in vitro* transendothelial electrical resistance measurements showed that this peptide destabilized endothelial barrier properties, and dampened the cAMP-mediated endothelial barrier stabilization induced by forskolin and rolipram. Immunofluorescence analysis revealed that TAT-Ahx-AKAPis led to both adherens junctions and actin cytoskeleton reorganization. Those effects were paralleled by redistribution of PKA and Rac1 from endothelial junctions and by Rac1 inactivation. Similarly, membrane localization of AKAP220 was also reduced. In addition, depletion of either AKAP12 or AKAP220 significantly impaired endothelial barrier function and AKAP12 was also shown to interfere with cAMP-mediated barrier enhancement. Furthermore, immunoprecipitation analysis demonstrated that AKAP220 interacts not only with PKA but also with VE-cadherin and ß-catenin. Taken together, these results indicate that AKAP-mediated PKA subcellular compartmentalization is involved in endothelial barrier regulation. More specifically, AKAP220 and AKAP12 contribute to endothelial barrier function and AKAP12 is required for cAMP-mediated barrier stabilization.

## Introduction

The vascular endothelium lining the intima of blood vessels precisely regulates the passage of solutes, macromolecules, and leukocytes between the blood and the underlying tissue. Under inflammatory conditions, mainly in post-capillary venules, loss of this primary function leads to formation of intercellular gaps and increased vascular permeability. The latter is a hallmark of several pathological processes (i.e. inflammation, allergy, arteriosclerosis, edema, tumor growth and sepsis) and contributes to multi-organ failure and death [1], [2]. Therefore, understanding of the mechanisms maintaining endothelial barrier functions under resting conditions, as well as the signaling pathways leading to barrier impairment or recovery are of great biological and clinical importance.

Paracellular permeability is tightly regulated by coordinate opening and closing of mainly two types of endothelial cell-cell junctions, namely tight junctions (TJs) and adherens junctions (AJs). While TJs seal the intercellular cleft between cells, the AJs are providing mechanical strength. However, the junctional composition of intracellular clefts varies across the vascular tree [3]. Both junctional types are composed of transmembrane proteins, i.e. the tight junction protein claudin-5 and the adherens junction protein VE-cadherin. These junctional markers are associated with the cortical actin cytoskeleton via several adaptor molecules such as zonula occludens (ZO) proteins and catenins, respectively [4]. Numerous studies showed that modulation of endothelial barrier functions via actin cytoskeleton remodeling and cell junction integrity can be controlled by members of the Rho family of small GTPases, i.e. RhoA, Rac1 and Cdc42 as well as by the Ras family GTPase Rap1. Although it is suggested that fine balance between activation and/or inactivation of these small GTPases is required for barrier maintenance, it is generally assumed that activation of RhoA impairs barrier function, while Rac1 and Cdc42 are considered to primarily stabilize barrier integrity [1], [5], [6], [7]. It is now widely recognized that a number of barrier-stabilizating mediators activate Rac1 either directly or indirectly via an increase in the concentration of the cellular second messenger cAMP. cAMP- dependent Rac1 activation can be achieved by both, exchange protein activated by cAMP1 (Epac1)/Ras-related protein 1 (Rap1), and cAMP-dependent protein kinase A (PKA) signaling pathways. The latter is generally believed to be the predominant cAMP mechanism that exerts significant protection against the increase in endothelial paracellular permeability [8]. Furthermore, it is assumed that precise spatiotemporally regulated activation is essential for the response specificity of the PKA pathways. Thus, it was found that a key role in tight regulation and compartmentalization of PKA-dependent signaling is played by A kinase-anchoring proteins (AKAPs) [9]. AKAPs are a large structurally diverse family of functionally related proteins that contain a conserved amphipathic helix PKA binding motif and function to localize PKA-AKAP complexes at discrete compartments within the cell such as plasma membrane, endoplasmic reticulum, mitochondria or Golgi complex [10]. By anchoring the inactive PKA to defined cellular sites, AKAPs allow specific placement of the holoenzyme at regions of cAMP production and thus to propagate confined phosphorylation of only a subset of potential substrates located in close proximity. AKAPs are also scaffolding proteins tethering not only PKA, but also other molecules involved in cAMP signaling such as adenylyl cyclases (ACs), phosphodiesterases (PDEs), Epac1, which is guanine nucleotide exchange factor (GEF) of Rap1 and protein phosphatases [11], [12], [13], [14], [15], [16]. Thus, AKAP complexes assemble PKA with a determined set of signal transduction (i.e. kinases) and termination (i.e. phosphatases, phosphodiesterases) molecules as well as with a variety of other members of different signaling pathways. Therefore, AKAPs organize crosstalk across diverse paths in the cell’s signaling networks [9], [17].

Although the protective effects of cAMP/PKA signaling for endothelial barrier regulation are well recognized, it is not yet clear by which mechanisms PKA is located close to cell junctions. Based on our previous investigations, we speculated that compartmentalized cAMP-signaling by AKAPs contribute to endothelial barrier integrity. Thus, we investigated the importance of AKAP function for maintenance of the cAMP/PKA-dependent endothelial barrier *in vivo* and *in vitro*.

In order to modulate AKAP function, we used a modified analog of a cell-permeable synthetic peptide designed to competitively inhibit PKA-AKAP interaction [18]. This peptide, named TAT-Ahx-AKAPis, is comprised of two functional peptides, TAT and AKAPis, connected via an aminohexanoic (Ahx) linker. AKAPis is a precisely designed sequence with high-affinity binding and specificity for the PKA regulatory subunit (RII) which enables a higher dissociation effect on the PKA-AKAP anchoring than the widely used Ht31 synthetic peptides [18]. The second functional unit, commonly denoted as TAT, is a cell-penetrating peptide derived from the TAT protein of human immunodeficiency virus (HIV-1). The TAT peptide possesses a high ability to mediate the import of membrane-impermeable molecules such as DNA, RNA, peptides and even whole proteins into the cell [19].

Although approximately 50 AKAPs have been identified in different cell types, little is known about the AKAP expression profile and function in endothelial cells. In the current investigation, besides AKAP12, which has already been found in endothelium and its involvement in regulation of endothelial integrity has been reported [20], [21], [22], [23], we focused on AKAP220. The latter was recently shown to contribute to the integrity of the cortical actin cytoskeleton [24], but was also suggested to link cAMP signaling to cell adhesion [25]. Both AKAP220 and AKAP12 are expressed in endothelial cells according to microarray data published in GeneCards database (http://www.genecards.org/).

In this study, by using *in vivo* and *in vitro* techniques, we provide evidence that AKAP-mediated PKA subcellular compartmentalization contributes to endothelial barrier integrity. Our data furthermore suggest AKAP220 and AKAP12 to be involved in these processes.

## Materials and Methods

### Cell culture

Human Dermal Microvascular Endothelial Cells (HDMECs) were obtained from PromoCell (Heidelberg, Germany). The cells were grown in Endothelial Cell Growth Medium MV containing supplement mix provided by the same company. Passage of the cells was performed by using Detach kit (PromoCell). All experimental procedures were carried out with HDMEC from passage 2 to 5 [26]. Additionally, an immortalized microvascular endothelial cells (MyEnd), previously isolated from murine myocardial tissue were used for the experiments. Cell generation and characterization have been described elsewhere [27], [28]. Cells were cultured in DMEM (Life Technologies, Karlsruhe, Germany), supplemented with Penicillin G/Streptomycin (50 U/ml) (Sigma-Aldrich, Munich, Germany) and 10% Fetal Calf Serum (FCS) (Biochrom, Berlin, Germany).

Both cell types were cultured at 37°C in a humidified atmosphere of 5% CO_2_.

### Antibodies and test reagents

The polyclonal rabbit anti-PKA RII alpha (sc-908) and the goat anti-VE-cadherin (sc-6458) antibodies were purchased from Santa Cruz Biotechnology (Santa Cruz, U.S.A.). Detection of VE-cadherin in MyEnd cells was performed by using rat anti-VE-cadherin mAb (clone 11D4.1; undiluted hybridoma). The mouse monoclonal anti-PKA RII beta (Cat.# 610625) and anti-ß-catenin (Cat.# 610154) antibodies were obtained from BD Biosciences (Franklin Lakes, U.S.A.). The mouse monoclonal anti-AKAP12 antibody (ab49849) was acquired from Abcam (United Kingdom). The rabbit polyclonal anti-AKAP220 antibody was kindly provided by John Scott (Howard Hughes Medical Institute, Department of Pharmacology, University of Washington School of Medicine).

To increase cAMP levels, Forskolin (F), (Cat.# F6886) and Rolipram (R), (Cat.#R6520) purchased from Sigma-Aldrich (St. Louis, U.S.A.) were used in combination for 1 hour at concentrations of 5 and 10 µM, respectively. In addition, cell-permeable synthetic peptide TAT-Ahx-AKAPis (GRKKRRQRRRXQIEYLAKQIVDDNAI) was utilized to competitively inhibit the interaction between the PKA regulatory subunit II (RII) and AKAPs. By using the ECIS system, preliminary concentration- effect experiments determined the effectiveness of the peptide on endothelial barrier stability (data not shown). The analysis revealed that 30 µM inhibitory peptide, dissolved in sterile distilled water with 10% DMSO, is the most effective peptide concentration for modification of endothelial barrier integrity. In parallel, experiments were conducted with TAT-Ahx-mhK77 scrambled synthetic peptide (GRKKRRQRRRXQFSSQSAFSSRSRRARS). The latter is similar to the inhibitory peptide regarding molecular weight, isoelectric point and amino acid composition. Both peptides were synthesized by Peptide Specialty Laboratories GmbH (Heidelberg, Germany). Simultaneously, a control condition (vehicle) was run. This internal control is composed of medium containing DMSO in a concentration corresponding to the one used for dissolving the peptides.

### Rac1 activation assay

To quantify the levels of Rac1 activity in cells, a Rac G-Lisa Activation Assay Biochem Kit (Cytoskeleton, Denver, CO; Cat.#BK126) was used. Briefly, confluent monolayers of HDMEC and/or MyEnd cells were incubated either in the absence or the presence of TAT-Ahx-AKAPis. Parallel administration of scrambled TAT-Ahx-mhK77 peptide was carried out. Besides F/R application, vehicle was applied as an additional control. Cells were lysed and the lysates were processed according to the manufacturer’s instructions. The absorption was measured at 490 nm using a TECAN, Infinite 200 PRO microplate reader (Tecan, Austria).

### Measurement of transendothelial resistance (TER)

An ECIS Z Theta system (Applied Biophysics Inc, Troy, U.S.A.) was used to assess the endothelial barrier integrity of confluent and subconfluent cell monolayers as previously described [29]. In short, the cells were grown to confluency on gold microelectrodes 8W10E+ (Ibidi, Germany, Cat.# 70040). MyEnd were seeded on gelatin-coated gold electrodes, HDMEC were grown on uncoated arrays. HDMEC cells reached confluency in between 8 to 10 days, while MyEnd formed a confluent monolayer within 3 to 4 days. Directly before the experiment, the medium was exchanged and the arrays were mounted onto the holders of the ECIS system, placed in an incubator (37°C, 5% CO_2_). For both cell types, the optimal frequency to analyze the changes in the transendothelial resistance was identified as 4000 Hz. After short equilibration for approximately 15 to 20 min, the baseline resistance was recorded for another 15 min, followed by application of the test reagent to each well yielding a final well volume of 400 µl.

For some experiments, the effect of AKAPs on endothelial barrier formation was tested by TER measurements initiated in subconfluent cell monolayers transiently transfected with specific siRNA. Cell confluency was determined by a light microscopy, subsequently the basal transendothelial electrical resistance was measured by ECIS system. Subconfluent MyEnd cells had a basal resistance of 550 to 800 ohm. The resistance monitored in confluent cell monolayers was above 800 ohm and reached values of approximately 1400 ohm.

### Immunoprecipitation (IP) and Western blot (WB) analysis

MyEnd cells were grown to confluence. After quick washing with ice-cold PBS, the cells were incubated for 10 min with pre-chilled precipitation buffer (0.5% Nonidet-40, 10 mM HEPES (pH 7.9), 1.5 mM MgCl_2_, 10 mM KCl, 5 mM EDTA), to which cOmplete protease inhibitor cocktail (Cat.# 11697498001, Roche) was freshly added. Cells were scraped and the homogenized suspension was clarified by centrifugation at 6000 g for 5 min, at 4°C. The supernatant was transferred to a fresh reaction vial and the protein concentration was estimated using the Bradford assay [30]. For immunoprecipitation with either rabbit anti-PKA or goat anti-VE-cadherin antibodies, cell lysates with 400–600 µg of total protein amount were pre-cleaned (for 1 hour at 4°C) by incubation with Protein G Plus-Agarose (Calbiochem Merck, Darmstadt, Germany Cat.#IP08). The resulting supernatant was incubated for 3 hours at 4°C with 1, 5 µg of defined antibody. To each sample, 25 µl of beads were added and overnight incubation at 4°C on a rocker platform was carried out. The beads with attached immunoprecipitated complexes were collected by centrifugation at 6000 g for 2 min, washed 3 times with cold washing buffer (0,05% Nonidet-40, 10 mM HEPES (pH 7.9), 1.5 mM MgCl_2_, 10 mM KCl, 5 mM EDTA) and resuspended in Laemmli buffer. Boiling at 95°C for 5 min serves to denature proteins and detach them from the beads. The immunoprecipitated complexes were separated on a SDS-PAGE gel, transferred to a nitrocellulose membrane and analyzed by Western blot (WB) assay [29]. The AKAP220 antibody was used in 1∶500 dilution, detection of AKAP12 was done with antibody diluted 1∶5000. All other antibodies were used in 1∶1000 dilution. Secondary antibodies were diluted 1∶3000.

ImageJ software was used to quantitatively assess the Western blot data. Same size rectangular areas were drawn around each band of interest and the signal intensity within the area was measured. Similarly, the fluorescence intensity determined at identically sized rectangles surrounding regions under or above the bands served as background. For the immunoprecipitation experiments, subtraction of the intensity determined within the control IP (IP: Beads, no Ab) was performed as well. Then the signal intensity was normalized either to the fluorescence intensity of housekeeping proteins (loading control) or to the intensity of the protein used to pull down desired protein complex. In order to combine all experiments, the data were presented as percent of control.

### Immunofluorescence (IF) analysis

Detailed description of the assay was previously published [29]. In short, HDMEC were cultured to confluency on uncoated glass cover slips. Control cells or cells treated with different test reagents as specified below were washed with PBS, fixed with 2% formaldehyde for 10 min at room temperature (RT) and, after washing with PBS, were permeabilized with 0,1% Triton X-100 in PBS. The adherent cell monolayer was blocked for an hour with 10% normal goat or donkey serum in 1% BSA/PBS followed by overnight incubation at 4°C with primary antibodies. After washing with PBS, the respective secondary antibodies conjugated with Cy3 (1 hour, RT) were applied. In addition, for visualization of filamentous actin, 1∶100 diluted ALEXA488-conjugated–phalloidin was added to the secondary antibody. Images were obtained by using a confocal microscopy setup (Leica SP5, Mannheim and Wetzlar, Germany) equipped with a HCX PL APO Lambda blue 63x 1.4.oil objective (Leica), with the same settings for all conditions tested.

Quantification of protein distribution was carried out using ImageJ computer software. For intensity determination, a rectangle was drawn over more than 15 randomly chosen areas of HDMEC plasma membrane in at least three independent experiments. The mean intensity distribution of proteins examined was recorded for each depicted rectangle. A region next to the cell without specific fluorescence was selected as the respective background. In order to combine and compare all immunofluorescence experiments, the data was presented as percent of control (vehicle).

### Transfection with small interfering RNA (siRNA)

Down-regulation of mouse specific AKAP12- and AKAP220 mRNA was obtained by using ON-Target SMARTpool siRNA (Dharmacon Thermo Fisher Scientific, Waltham, U.S.A.; Cat.# L-046094-00-0005371,00 and Cat.#L-048015-00-0005, respectively). As a negative control, ON-TARGET plus Non-Targeting siRNAs (Dharmacon, Cat.# D-001810-10-20) was applied. The siRNA was delivered into the cells by applying TurboFect *in vitro* transfection reagent (Fermentas, St. Leonrot, Germany, Cat.# R0531). The transient transfection was carried out according to the manufacturer’s protocol. Briefly, after 20 min pre-incubation at RT, the transfection solution composed of TurboFect, siRNA and serum- free DMEM was diluted into serum-containing medium and added dropwise to MyEnd cells with 60–70% confluence. 48 hours after transfection, AKAPs-depletion was confirmed by Western blot.

For ECIS-based measurements, MyEnd were transfected with specific siRNA at 70% confluency. 24 hours after transfection medium was exchanged and TER was monitored. Basal and cAMP- stimulated Rac1 activities were examined 48 hours after siRNA transfection in control cells or cells treated with F/R, respectively.

### Animal preparation and measurement of hydraulic conductivity (L_p_) of the microvessel wall

A detailed description of the animal preparation and the microvessel L_p_ measurements was reported elsewhere [27], [31]. All experimental protocols and procedures were consistent with the requirements of the National Institute of Health “Guide for the Care and the use of Laboratory Animals” and approved by Government of Lower Franconia (Az 55.2-2531.01-24/09). Wistar rats (Charles River Laboratories, Sulzfeld, Germany), with body weight ranging from 250 to 450 g, were anesthetized by subcutaneous injection of pentobarbital sodium at a dose of 65 mg/kg. The anesthetic substance and its way of application were chosen not to interfere with blood vessel permeability. In addition, depth of anaesthesia was checked regularly by animal’s reaction to foot pad pinching. Supplemental anaesthetic was given only if the above mentioned reaction was positive. The experiments were carried out using straight, non-branched segments of mesenteric venular microvessels (25 to 35 µm in diameter). As descried earlier, the L_p_ measurements of the microvessel wall are based on the modified Landis technique, which measures the volume flux of fluid per unit surface area of the vessel (*J_v_/S*), which was canulated with a glass micropipette and occluded in advance [32], [33]. During measurements, the hydraulic pressure of usually 50 cm H_2_O was constant with the assumption that the net effective pressure determining fluid flow (Peff) was equal to the applied hydraulic pressure minus 3 cm H_2_O (the approximate oncotic pressure contributed by BSA in all perfusates (10 mg/ml)) [27]. For each occlusion, *L*_p_ was estimated as (*J*_v_/*S*)/Peff. All perfusates were mammalian Ringer’s solution containing 10% BSA (Sigma-Aldrich) with or without TAT-Ahx-AKAPis inhibitory synthetic peptide. Measurements were performed every 10 min for up to 120 min in the presence and absence of the synthetic peptide. Each animal was used for a single experiment only. After experimental completion, the unconscious rats were euthanized by intracardial injection of saturated KCl solution.

### Statistical analysis

The data were processed using Prism Software version 5 (Graph Pad, San Diego, U.S.A.). All values are expressed as means ± SEM. While 2-tailed paired Student’s t-test was used to compare two groups, One-way Analysis of Variance (ANOVA) followed by a Tukey’s or Bonferoni’s Multiple comparison tests were applied to determine the differences among multiple groups. The results were considered statistically significant at p≤0.05.

## Results

### TAT-Ahx-AKAPis peptide impaired endothelial barrier properties *in vitro*

**Figure 1:**
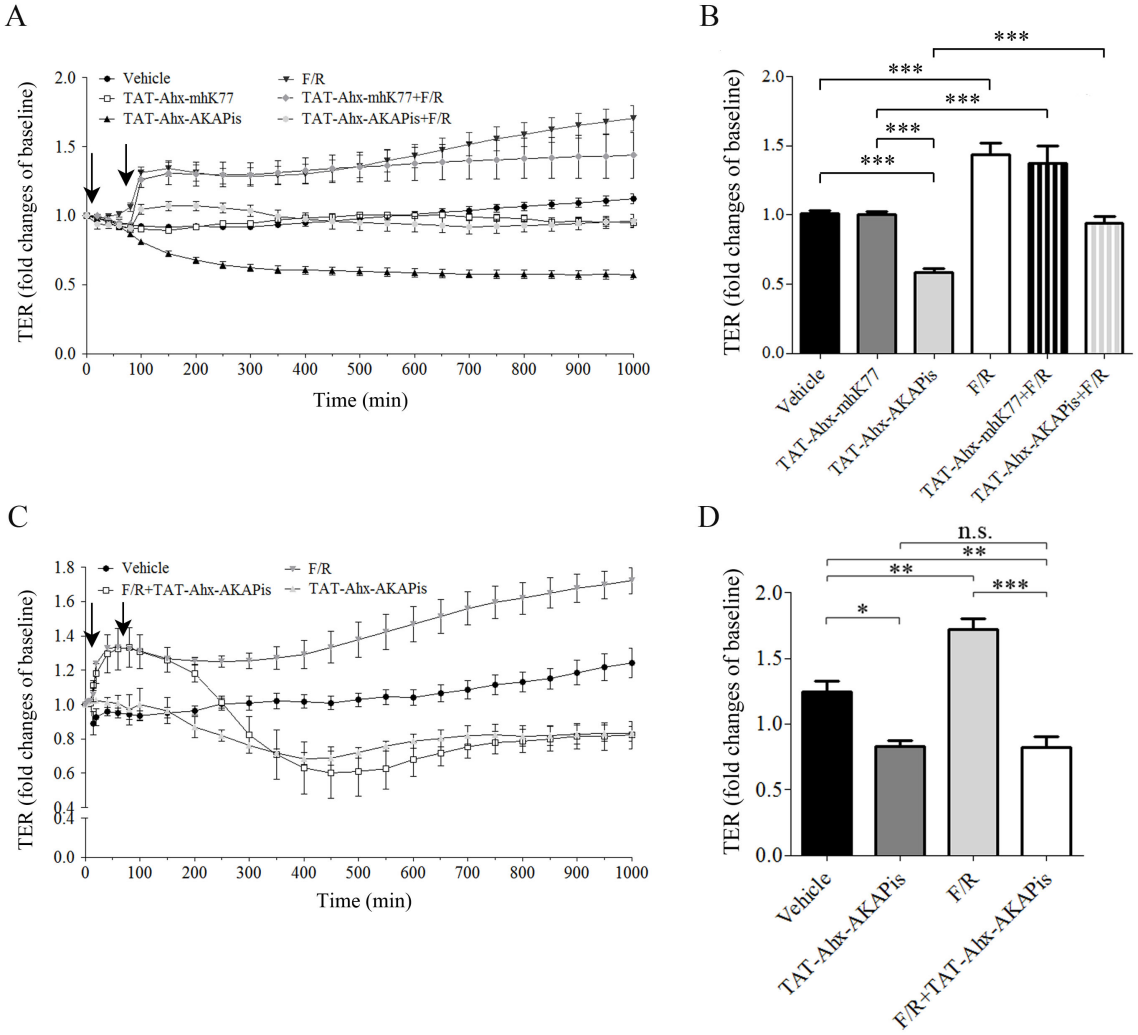
TAT-Ahx-AKAPis significantly reduced microvascular endothelial barrier function and reverted F/R- mediated barrier stabilization. TER measurements were carried out to monitor endothelial barrier alterations in response to different mediators and synthetic peptides. (A) displays the time course of TER measurements under various experimental conditions for HDMEC. The first arrow indicated the time point of TAT-Ahx-AKAPis and/or TAT-Ahx-mhK77 addition. 1 hour after the first application, F/R was added (second arrow). (B) summarizes the results after 600 min, the time point at which the monitored effects reached their peaks. TAT-Ahx-AKAPis significantly decreased TER compared to control/vehicle condition and treatment with scrambled peptide (TAT-Ahx-mhK77) starting at 80 min after application for HDMEC. F/R addition resulted in pronounced and continuous increase of TER after 1 hour. A similar effect was detected after pre-incubation with TAT-Ahx-mhK77 scrambled peptide. In contrast, 1 hour pre-treatment with TAT-Ahx-AKAPis initially reduced, but subsequently did not abolished the effect of F/R. (C) To further test the effect of TAT-Ahx-AKAPis on F/R- mediated enhancement of endothelial barrier function, HDMEC cells were exposed to F/R for 1 hour (first arrow) and post- incubated with TAT-Ahx-AKAPis inhibitory peptide (second arrow). (D) graphically represents the statistical outcome of the data presented in panel (C). Based on ANOVA multiple analysis, the most significant peaks of the monitored effects were determined at 1000 min. At that time point (1000 min), the similarly responding TAT-Ahx-AKAPis- and F/R+TAT-Ahx-AKAPis- cell monolayers displayed TER significantly lower than the one in control monolayers. In contrast, F/R- mediated enhancement in TER remained constant over time. Data were collected from more than three independent experiments (N ≥3, n≥4–10). *** p≤0.001, ** p≤0.01, * p≤0.05, indicate statistically significant difference between examined groups. n.s. – not significant.

To study the role of AKAP-mediated PKA subcellular anchoring for maintenance of endothelial barrier properties, we used a cell-permeable synthetic peptide (TAT-Ahx-AKAPis) designed to disrupt the endogenous PKA-AKAP complex [18]. Confluent microvascular endothelial cells were treated with TAT-Ahx-AKAPis (30 µM) and subjected to continuous TER measurements. In HDMEC, application of TAT-Ahx-AKAPis significantly decreased TER after 80 min incubation (80.4±2.04% of baseline values) compared to control conditions (vehicle: 92±1.2% of baseline (p≤0,01) and TAT-Ahx-mhK77∶90±1.93% of baseline (p≤0,05) (Figure 1, A). Microvascular murine MyEnd cells responded similarly but slower to the inhibitory peptide (Figure S1). Nonetheless, in both cell types, TAT-Ahx-AKAPis induced a slowly progressive decrease in TER. In HDMEC, after 600 min of application, values reached 58±3%, whereas the values in control cells treated with vehicle remained stable (101±2.4%) (Figure 1, A - B). In contrast, treatment with TAT-Ahx-mhK77 did not affect TER in HDMEC (100±2.0%) (Figure 1, A - B).

The AC activator forskolin and the PDE IV inhibitor rolipram (F/R) increase cAMP levels and subsequently activate Rac1, the latter was found to be at least in part required for maintenance of endothelial barrier properties [7], [29], [34], [35]. Thus, in line with previous studies [29], [36], treatment with F/R (5 µM/10 µM) increased TER within 1 hour in both cell lines, with the effect being more prominent in HDMEC. Within the first hour, F/R rapidly increased TER in HDMEC (139±5.6%) and remained constantly elevated while in controls no significant changes were detected (92±1.2%). After 600 min, F/R reached its maximal effect compared to control conditions with values being 143±8.4% (Figure 1, A–B). Interestingly, in contrast to the pretreatment with scrambled peptide (TAT-Ahx-mhK77), where no significant alterations in the F/R effect (detected in control conditions) were observed, pretreatment with the TAT-Ahx-AKAPis inhibitory peptide diminished the F/R-mediated increase in TER relative to control levels. This effect of TAT-Ahx-AKAPis was also clearly detectable in MyEnd cells but again was evident later (Figure S1). To further evaluate the effect of TAT-Ahx-AKAPis on F/R- mediated endothelial barrier stabilization, HDMEC monolayers treated for 1 hour with F/R were subsequently subjected to TAT-Ahx-AKAPis peptide (Figure 1, C). The analysis revealed that 1 hour after TAT-Ahx-AKAPis application TER was similar in cells treated with F/R+TAT-Ahx-AKAPis or F/R alone (F/R+TAT-Ahx-AKAPis: 126±7%; FR: 127±1%). However, starting 100 min after peptide addition TER decline and after 230 min was comparable to experiments in which cells were treated with TAT-Ahx-AKAPis alone (at 300 min: F/R+TAT-Ahx-AKAPis: 82.4±10.7%; TAT-Ahx-AKAPis: 76±1.4%, Figure 1, C). Thus, the most significant peaks of the monitored effects were determined at 1000 min, the time point where similarly responding TAT-Ahx-AKAPis and F/R+TAT-Ahx-AKAPis monolayers displayed TER significantly lower than that of the control cells. In contrast, treatment of HDMEC with F/R significantly increased TER compared to controls as TER remained continuously elevated during the entire experiment (Figure 1, D).

Taken together, these results indicate that TAT-Ahx-AKAPis was sufficient to disrupt microvascular endothelial barrier properties, presumably via preventing AKAP-PKA complexation. Additionally, post-treatment with TAT-Ahx-AKAPis reverted F/R- mediated barrier stabilization whereas the pretreatment with the synthetic peptide was uneffective to completely abolish the barrier enhancing effect of F/R.

### TAT-Ahx-AKAPis-induced endothelial barrier disruption was paralleled by VE-cadherin reorganization and actin cytoskeleton remodeling

**Figure 2:**
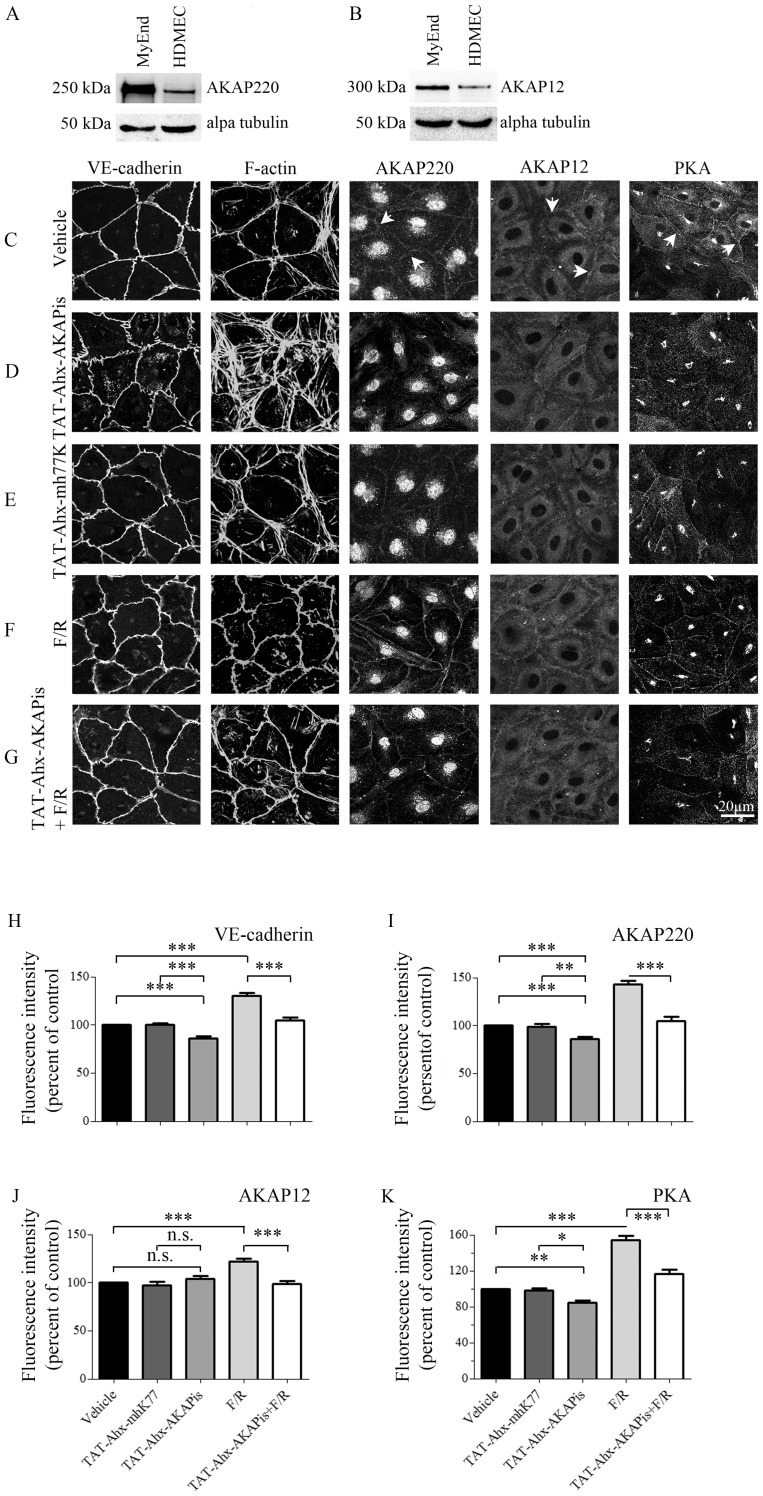
Effects of PKA compartmentalization on adherens junctions, actin cytoskeleton and AKAP organization. To determine the AKAPs protein expression profile, equal amounts of MyEnd and HDMEC cell lysates (20 µg per line) were subjected to WB analysis. The expression patterns of AKAP220 (A) and AKAP12 (B) were analyzed. Loading was controlled by alpha tubulin expression. Data are representative of three or more independent experiments (N≥3). In addition, HDMEC monolayers were treated for 1 hour either with vehicle, TAT-Ahx-AKAPis, TAT-Ahx-mhK77, F/R or with a combination of 1 hour TAT-Ahx-AKAPis pretreatment followed by 1 hour F/R application. The distributions of VE-cadherin, PKA, AKAP220 and AKAP12 were assayed by immunofluorescence. Additionally, ALEXA-488-conjugated phalloidin was used for visualization of F-actin. (C) Under control condition, VE-cadherin displayed slightly interdigitated but continuous staining along cell borders, and the actin cytoskeleton was preferentially organized cortically. The staining of AKAP220, AKAP12 and PKA was detectable at cell borders (arrows). (D) In clear contrast, exposure to TAT-Ahx-AKAPis increased interdigitations and significantly reduced the intensity of VE-cadherin staining. Profound reorganization of the actin cytoskeleton was paralleled by substantial reduction of AKAP220 and PKA, but not of AKAP12 membrane staining. (E) However, cell monolayers incubated with TAT-Ahx-mhK77 showed immunofluorescence staining similar to control for all proteins under investigation. (F) Not surprisingly, F/R treatment resulted in pronounced and linearized VE-cadherin appearance, intensified cortical actin cytoskeleton, and pronounced membrane staining for AKAP220, AKAP12 and PKA compared to control conditions. (G) 1 hour pre-incubation with TAT-Ahx-AKAPis followed by 1 hour treatment with F/R resulted in monolayers largely similar to controls, but not to F/R incubation alone. Images are representative of three or more independent experiments (N ≥3). Scale bar = 20 µm. The above presented data were confirmed by quantification of signal intensity distribution at cell borders. (H–K) demonstrates the mean intensity peak observed at cell borders (n≥15). *** p≤0.001, ** p≤0.01, * p≤0.05, indicate statistically significant difference between examined groups; n.s., not significant.

Endothelial barrier functions are dependent on the organization of junctional complex and the actin cytoskeleton [35]. Therefore, possible alterations of these structures accompanying the TAT-Ahx-AKAPis-induced decrease in TER were investigated by immunofluorescence studies in HDMEC. Subsequently, measurements of the fluorescence intensity along cell borders served to quantitatively assess changes in the distribution of membrane associated proteins. Beside the adherens junction protein VE-cadherin, filamenteous (F-) actin, and PKA, stainings for AKAP12 and 220 were also performed. The AKAP 12 and 220 expression profiles were initially evaluated by Western blot in human and mouse microvascular endothelial cells (Figure 2, A and B). The analysis revealed more prominent expression of AKAPs in MyEnd cells.

HDMEC monolayers were treated for 1 hour either with vehicle solution, TAT-Ahx-AKAPis, corresponding scrambled peptide or F/R. In addition, a regimen of 1 hour TAT-Ahx-AKAPis pretreatment followed by 1 hour F/R application was carried out. The cell monolayers incubated with vehicle solution displayed slightly interdigitated but continuous VE-cadherin staining along cell borders as well as few stress fibers localized predominantly in cortical regions (Figure 2, C). Peripheral membrane localization was partially visible for AKAP220, AKAP12 and PKA (Figure 2, C, arrows). However, when HDMEC were treated with TAT-Ahx-AKAPis, pronounced reorganization of the actin cytoskeleton accompanied by enhanced interdigitations and decreased staining intensity of VE-cadherin were detectable (Figure 2, D, H). This was paralleled by considerable reduction of PKA and AKAP220 but not AKAP12 membrane staining (Figure 2, D, I–K) indicating that at least in the case of AKAP220 the peptide was effective in disrupting PKA anchorage at sites of cell contacts. In contrast, the proteins under investigation showed distributions similar to controls when monolayers were treated with scrambled synthetic peptide (Figure 2, E, H–K). Compared to controls, as reported previously [26], [29], F/R treatment resulted in more intense and linearized VE-cadherin staining (Figure 2, F, H). Moreover, membrane staining for AKAP12, AKAP220 and PKA was also more pronounced (Figure 2, F, I–K). This was accompanied by intensified cortical actin staining (Figure 2, F). In good agreement with the TER data pre-incubation with the inhibitory peptide interfered with the initial effect of F/R. HDMEC monolayers appeared more similar to controls (Figure 2, G–K). In summary, the above presented data showed that TAT-Ahx-AKAPis induced reorganization of both endothelial adherens junctions and the actin cytoskeleton as well as caused AKAP220 and PKA relocation from the membrane.

**Figure 3:**
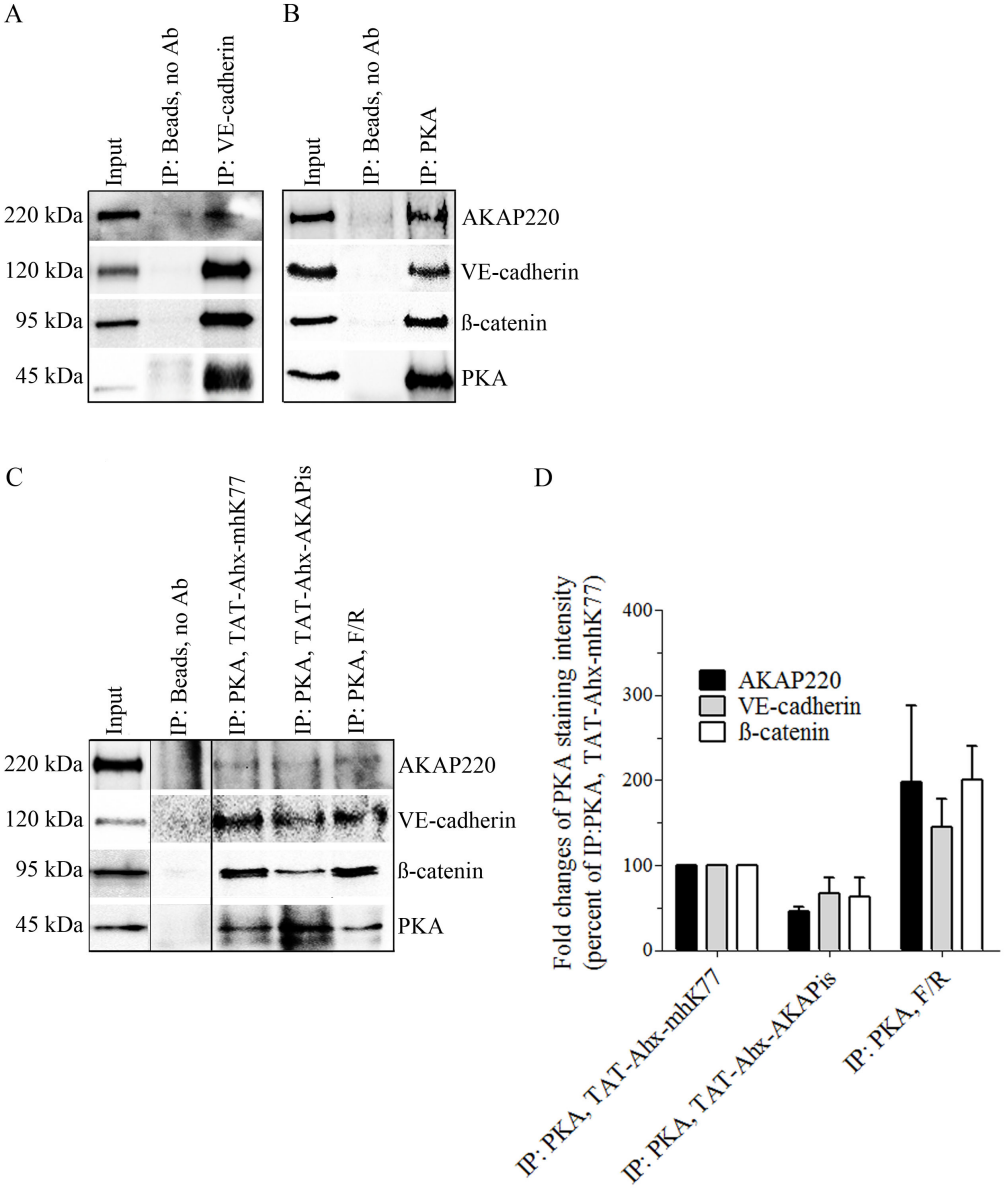
PKA and AKAP220 form a complex with junctional-associated proteins VE-cadherin and ß-catenin. A complex consisting of PKA, ß-catenin, VE-cadherin and AKAP220 was detected in MyEnd either by pulling down VE-cadherin (A) or PKA (B). Immunoprecipitated proteins and their binding partners were detected in the lysates used for immunoprecipitation. The lysates were loaded at the first lane and denoted as an “Input”. Immunoprecipitation without antibody (IP: Beads, no Ab) was used as a negative control. Every other lane represents proteins in complex under different experimental conditions. (C) To test the role of PKA compartmentalization on the stability of the determined complex, PKA pull down was performed in cells treated either with inhibitory or with the respective scrambled peptide. Additionally, the effect of F/R on complex stability was analyzed. (D) summarizes data collected from three independent immunoprecipitation experiments. Firstly, intensity readings for each band were background corrected. Secondly, data was modified by substraction of “non-specific background intensities” detected in IP without Ab (negative control). Since PKA was used to pull-down the complex, signal intensity of the immunoprecipitated proteins was normalized to the band intensity of PKA (represented as fold changes of PKA staining intensity). As a final, the data was presented as percent of IP: PKA, TAT-Ahx-mhK77. The analysis revealed decreased complex association of ß-catenin, VE cadherin and AKAP220 after TAT-Ahx-AKAPis treatment. In contrast, F/R application led to stabilization of the complex indicated by more prominent intensities for bands representing ß-catenin, VE-cadherin and AKAP220. Noncontiguous bands run on the same gel are separated by a black line. Images are representative of three independent experiments (N = 3).

In endothelial adherens junctions, VE-cadherin in addition to a variety of structural proteins (i.e. ß-catenin, p120-catenin and plakoglobin) associates with several molecules participating in cAMP signaling such as PKA, PDE IV and Epac1 [37]. On the other hand, it is well known that PKA is tethered by AKAP220 [38] and the latter was suggested to be connected to cytoskeletal structures [39]. Therefore, we speculated that PKA via AKAP220 interacts with junctional complexes which may be required for stabilization of the endothelial barrier. To test this hypothesis, MyEnd lysates were subjected to immunoprecipitation. The analysis confirmed a complex consisting of AKAP220, PKA, ß-catenin and VE-cadherin. Both, pulling down VE-cadherin or PKA, respectively, yielded the same results (Figure 3, A and B). In addition, to monitor the changes in the complex composition as a result of TAT-Ahx-AKAPis and/or F/R treatment, PKA pull-down in lysates derived from cells treated either with synthetic inhibitory peptide or with F/R was carried out. PKA pull-down in cells subjected to scrambled peptide was used as respective control. Compared to TAT-Ahx-mhK77 treatment, application of TAT-Ahx-AKAPis reduced the band intensities for AKAP220 as well as for VE-cadherin and ß-catenin indicating decreased association with PKA. In contrast, F/R enhanced ß-catenin-, VE-cadherin- and AKAP220- band intensities (Figure 3, C–D).

### AKAP12 and AKAP220 are involved in regulation of endothelial barrier function

**Figure 4:**
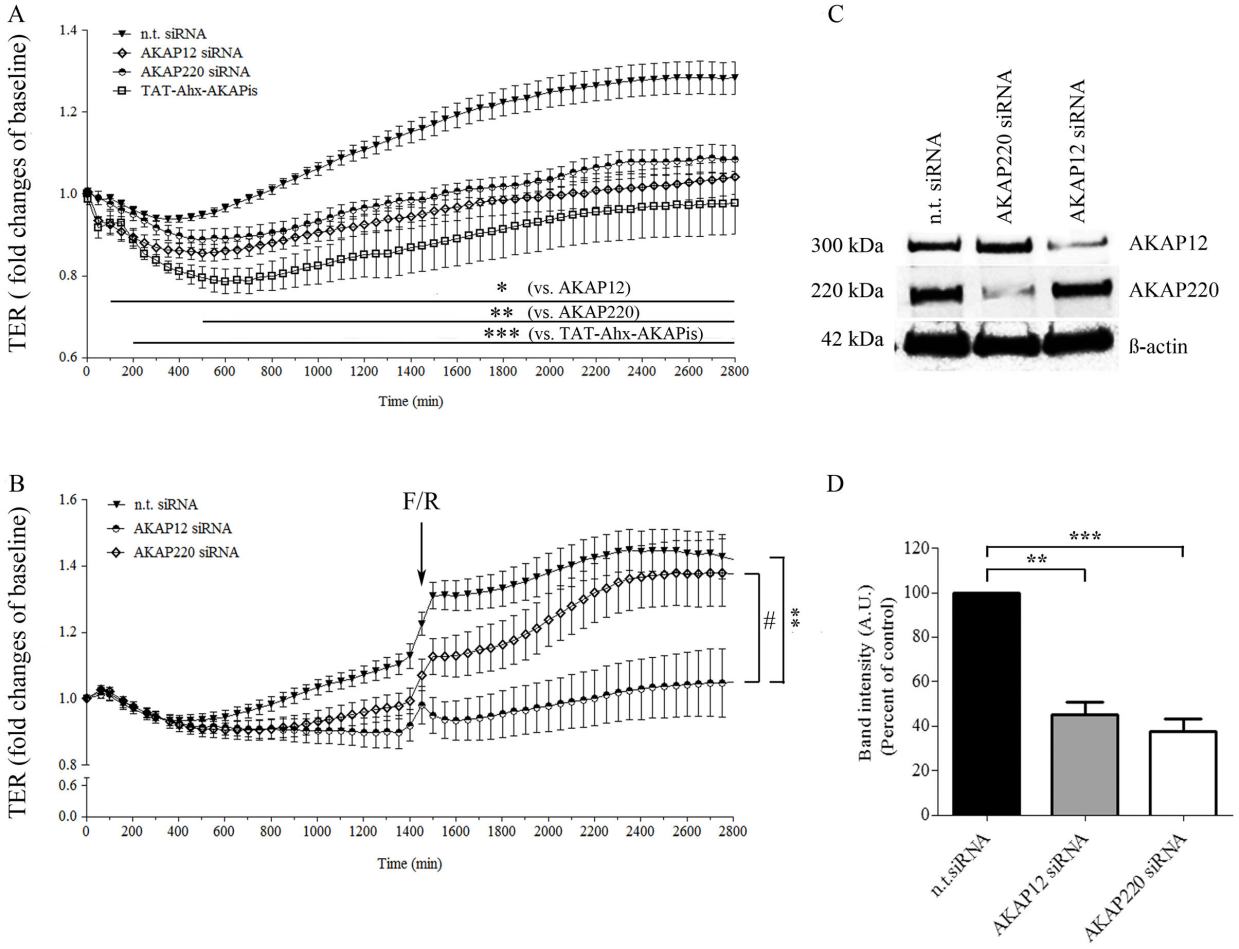
siRNA-mediated AKAP12 and AKAP220 knockdown significantly impaired endothelial barrier function. (A) Barrier function of subconfluent MyEnd cells was monitored by TER measurements following transfection with siRNA specific for AKAP12 and AKAP220. Non-targeting siRNA was used as a control. The results were compared to cells treated with TAT-Ahx-AKAPis inhibitory peptide. AKAP12 and AKAP220 depletion lead to a significant decrease in TER compared to monolayers transfected with n.t. siRNA. Similar, but more prominent, was the effect obtained after TAT-Ahx-AKAPis application. Data were collected from three or more independent experiments (N ≥3, n ≥12). (B) To test the role of specific AKAPs on cAMP-mediated endothelial barrier formation, TER measurements in cells treated with F/R, 48 hours after successful AKAP-depletion were performed; (n≥10). (C) AKAP12 and AKAP220 knockdown was confirmed by Western blot, 48 hours after transfection. ß-actin was used as an internal gel-loading control. (D) Western blot data were analyzed densitometrically. Normalized intensities for AKAP signals are presented as a bar graph. Data were collected from more than three independent experiments (N ≥3). * p≤0.05, **p≤0.01 and ***p≤0.001 indicate statistically significant difference between the examined group and n.t. siRNA; # p≤0.05 shows statistically significant difference between AKAP12 siRNA and AKAP220 siRNA upon treatment with F/R. The difference was significant starting at 1500 min.

To further investigate the role of AKAPs, the effect of AKAP220- and AKAP12- specific depletion on endothelial barrier function was determined and compared to treatment with TAT-Ahx-AKAPis. Subconfluent MyEnd cells were transiently transfected either with AKAP220- or AKAP12- specific siRNA or with n.t siRNA, respectively. 24 hours after siRNA application, TER measurements were initiated (Figure 4, A). The beginning of the TER measurements was also the initial point of TAT-Ahx-AKAPis peptide application. The experiments were continued for additional 46 hours. The time window was estimated by Western blot analysis validating the efficiency of the gene silencing in MyEnd treated with AKAP-specific siRNAs (Figure 4 C, D).

Control cells transfected with n.t. siRNA increased TER over time to values of 128.6±3.95% of baseline (Figure 4, A). In contrast, siRNA-mediated AKAP12 and AKAP220 knockdown initially decreased TER and subsequently abolished barrier stabilization (104±4.3% and 108±3.6%, respectively). Similar, but more significant was the effect upon TAT-Ahx-AKAPis inhibitory treatment (98±7.6%). Thus, these data indicate that besides AKAP12 and AKAP220 possibly other AKAPs are involved in the regulation of endothelial barrier function.

In order to estimate the effect on cAMP-mediated endothelial barrier function, F/R was applied to cells either transiently depleted of specific AKAPs or treated with n.t. siRNA (Figure 4, B). The results indicate that depletion of AKAP12, but not of AKAP220 significantly decreases the effect of cAMP-mediated endothelial barrier stabilization. These data suggest that both AKAPs alter endothelial barrier function but only AKAP12 modifies the subsequent cAMP-mediated endothelial barrier enhancement.

### Disruption of the PKA-AKAP endogenous complex reduced Rac1 activity

**Figure 5:**
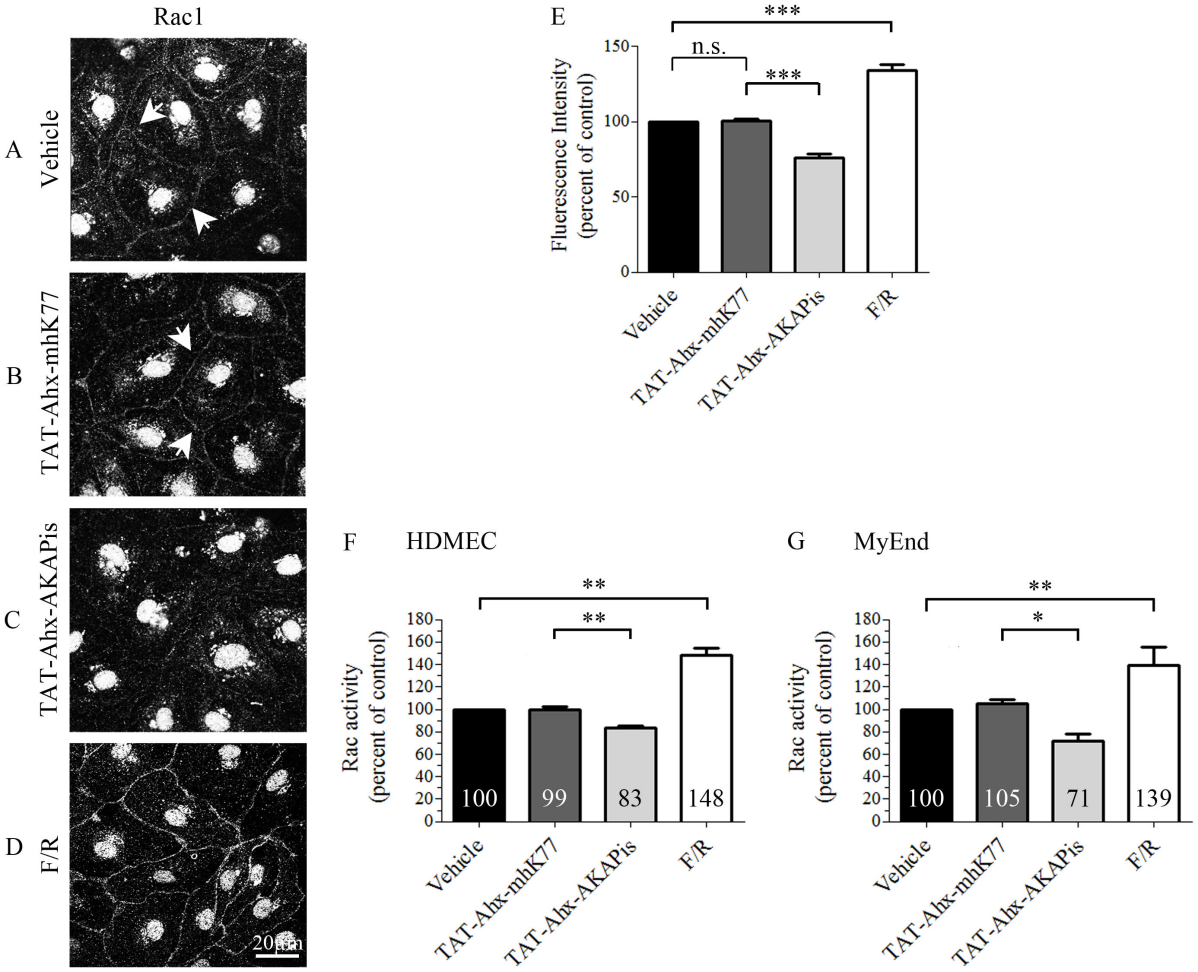
TAT-Ahx-AKAPis reduced Rac1 activity. HDMEC monolayers grown on glass coverslips were subjected to immunostaining with anti-Rac1 antibody. (A) In controls, Rac1 was partially present along cell borders (arrows). (B) Similar Rac1 staining was observed after application of TAT-Ahx-mhK77. (C) In contrast, treatment with TAT-Ahx-AKAPis strongly reduced Rac1 membrane staining and induced re-localization of the molecule to the cytoplasm. (D) However, the cAMP enhancers F/R led to pronounced and intensified Rac1 membrane staining. Images are representative of three or more experiments (N≥3). Scale bar = 20 µm. (E) Quantification of the signal intensity distributed along cell-cell junctions supported the above mentioned observations. In order to summarize all experiments, data were presented as percent of control (N≥3¸n≥10). (F) In HDMEC, ELISA-based measurements revealed a significant decrease of Rac1 activity in response to 1 hour treatment with TAT-Ahx-AKAPis. Application of F/R also significantly increased Rac1 activity when compared to control. As expected, TAT-Ahx-mhK77 administration had no effect on Rac1 activity. (G) The effects observed in MyEnd cells were similar. In agreement with TER, the effect of TAT-Ahx-AKAPis inhibitory peptide was evident later. Therefore, the MyEnd samples were collected 6 hours after peptide application. The results are representative of three independent experiments (N = 3, n ≥6). * defines statistically significant difference; * p≤0.05, ** p≤0.05, *** p≤0.001. n.s. not significant.

Our data demonstrate that TAT-Ahx-AKAPis-mediated disruption of the endogenous PKA–AKAP complex attenuated endothelial barrier functions under resting conditions. Since cumulative evidence shows that cAMP governs microvascular barrier properties, at least in part, in a Rac1-dependent manner [35], [40], [41], we investigated the effect of TAT-Ahx-AKAPis on Rac1 localization and activity. Immunofluorescence analysis in HDMEC revealed that, under control conditions, Rac1 staining was in part detectable along cell borders, (Figure 5, A, arrows). Such membrane localization of Rac1 was previously correlated with an increase in its activity. In this respect, our previous study showed that constitutively active Rac1 localized to cell- cell borders in endothelial cells whereas this effect was not observed in cells transfected with dominant negative Rac1 [42]. However, strong reduction of Rac1 membrane staining and relocation to the cytoplasm were detected after TAT-Ahx-AKAPis application (Figure 5, C). Further densitometric assessment of the immunofluorescent data confirmed these observations (Figure 5, E). Consistently, Rac1 rearrangement was paralleled by altered GTPase activity in HDMEC and MyEnd cells as measured by G-LISA Rac activation assay (Figure 5 F–G). However, treatment with TAT-Ahx-mhK77 neither showed changes in Rac1 localization nor in Rac1 activity when compared to control condition (Figure 5, B, E–G). In contrast, application of F/R dramatically enriched the staining of Rac1 at the membrane (Figure 5, D–E). Consistent with the immunofluorescence analysis, F/R caused a significant increase of Rac1 activity in both cell types (for HDMEC, F/R: 148±5% and for MyEnd, F/R: 139±14%; Figure 5 F–G). In HDMEC, the latter was approximately 48% more than the activity determined in controls or scrambled-treated cells (vehicle: 100%; TAT-Ahx-mhK77∶99±3%). The effect in MyEnd cells was similar, but slightly smaller (more than 33%), (vehicle: 100%; scrambled: 105±3% (n.s.)). ELISA-based Rac1 activity measurements also demonstrated that peptide-application significantly reduced Rac1 activity to 83±2% of control conditions in HDMECs and 71±6% in MyEnd cells (Figure 5 F–G).

**Figure 6:**
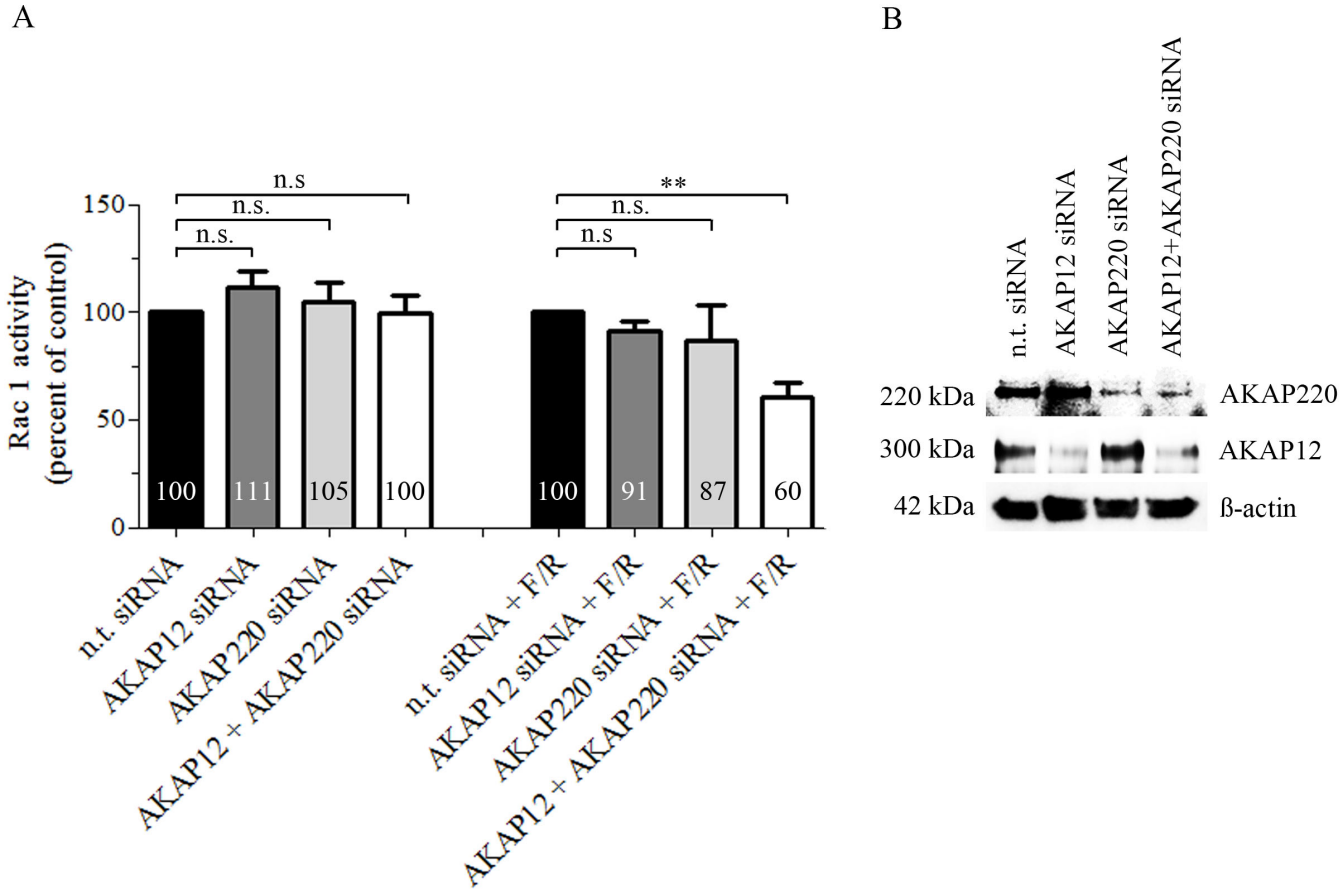
Simultaneous knockdown of AKAP12 and AKAP220 mRNA impaired cAMP-mediated Rac1 activation. (A) Subconfluent monolayers of MyEnd cells were transfected either with single AKAP siRNA or with a pool of AKAP12 and AKAP220 specific siRNAs. Basal Rac1 activity was tested 48 hours after initial siRNA depletion. For evaluation of cAMP-mediated Rac1 activation, transfected cells were subjected to F/R for 1 hour. Non-target siRNA (n.t. siRNA) alone or in combination with F/R (1 hour) was used as a respective control. Bars represent mean values ± SEM. Basal activity of Rac1 was unaffected after single or combined silencing of AKAPs. In contrast, cAMP-mediated Rac1 activation was significantly impaired following simultaneous AKAP12 and AKAP220 mRNA knockdown. (B) Successful knockdown was verified by Western blot 48 hours after initial siRNA transfection; ß-actin was used as an internal gel-loading control. ** indicates p≤0.01 versus cells transfected with n.t. siRNA treated for an hour with F/R.

To further evaluate the effect of specific AKAPs on Rac1 activity, we silenced AKAP12 or AKAP220 by siRNA and assessed Rac1 activity 48 hours after knockdown in MyEnd cells. Neither down-regulation of AKAP12 and/or AKAP220 mRNA alone nor parallel silencing of both AKAPs altered basal Rac1 activity (Figure 6, A). Nevertheless, cAMP-mediated Rac1 activation was significantly reduced in cells simultaneously depleted for AKAP12 and AKAP220 but not in cells in which only one of the two AKAPs was silenced (Figure 6, A). Effective mRNA down-regulation was validated by Western blot (Figure 6, B).

### TAT-Ahx-AKAPis treatment increased hydraulic conductivity (L_p_) of single perfused rat postcapillary venules *in vivo*

**Figure 7:**
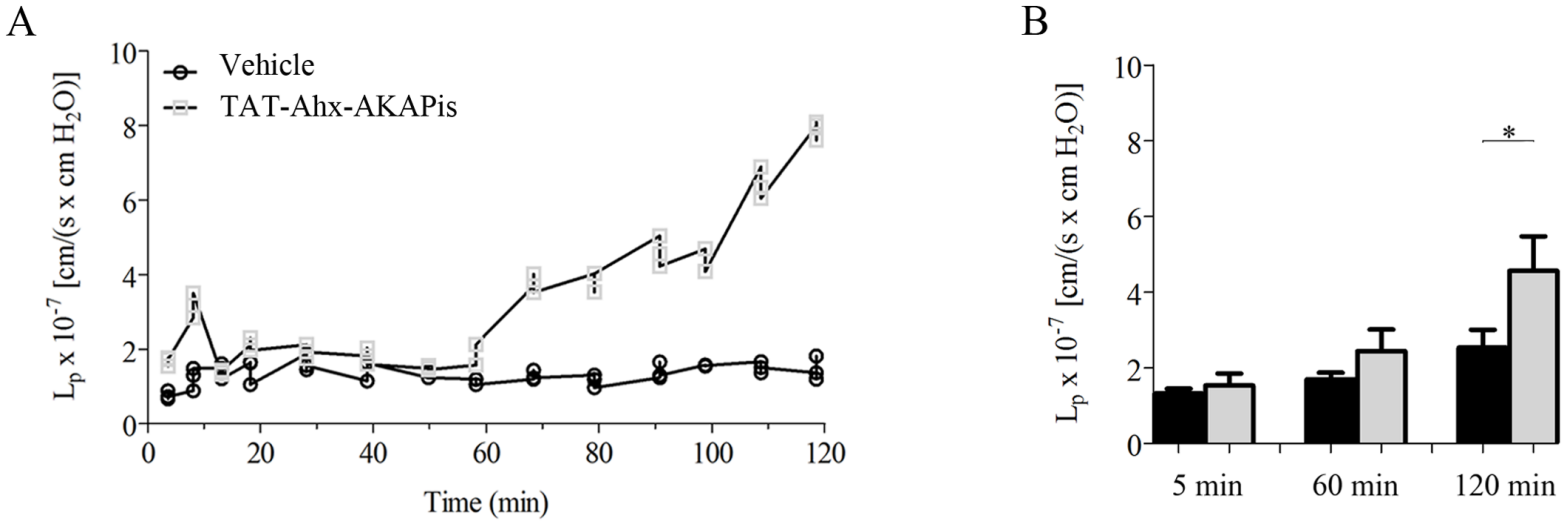
TAT-Ahx-AKAPis application increased hydraulic conductivity (L_p_) in rat mesenteric microvessels *in vivo.* Hydraulic conductivity (L_p_) of isolated post-capillary microvessels from rat mesentery was monitored over time. (A) displays data from a representative vessel constantly perfused either with vehicle or with TAT-Ahx-AKAPis. (B) Bar diagram summarizes the mean values of all five independent experiments. 120 min after TAT-Ahx-AKAPis perfusion, L_p_ was significantly increased to 4.57±0.91×10^−7^ [cm/(s×cm H_2_0)] compared to the corresponding vehicle condition (2.53±0.47×10^−7^ [cm/(s×cm H_2_0)]). *indicates p≤0.05 versus control condition; N = 5.

To investigate whether the application of TAT-Ahx-AKAPis also affects microvascular barrier properties *in vivo,* we applied the single microvessel perfusion technique [27] in rat mesentery postcapillary venules. Intact microvessels were constantly perfused either with TAT-Ahx-AKAPis or with vehicle solution and hydraulic conductivity was measured every 10 min. No significant L_p_ differences of TAT-Ahx-AKAPis-treated and control vessels were monitored within the first 60 min (Figure 7, A and B). However, L_p_ increased starting 60 min after TAT-Ahx-AKAPis addition. After 120 min of treatment, L_p_ of TAT-Ahx-AKAPis- perfused vessels reached a mean value of 4.57±0.91×10^−7^ [cm/(s×cm H_2_O)]) which was significantly higher compared to controls (2.53±0.47×10^−7^ [cm/(s×cm H_2_O)]), (Figure 7, B). Thus, TAT-Ahx-AKAPis impaired microvascular endothelial barrier properties both *in vivo* and *in vitro*.

## Discussion

The goal of the current report was to study in more details the mechanisms underlying cAMP-mediated regulation of endothelial barrier properties. Specifically, we examined the functional relevance of AKAPs which on one hand are well established to tightly regulate PKA function by facilitating its discrete cellular compartmentalization, and on the other hand are known to form multivalent complexes to integrate cAMP signaling with various other pathways. We provided evidence that AKAPs are required for maintenance of microvascular endothelial barrier integrity both *in vivo* and *in vitro* under resting conditions. We also showed that under resting conditions AKAPs are relevant for correct signal transduction because Rac1 activity was decreased following inhibition of PKA-AKAP anchoring. Particularly, we also found that AKAP12 and AKAP220 are involved in regulation of endothelial barrier function. Further examinations revealed that simultaneous down-regulation of AKAP12 and AKAP220 expression impaired F/R-mediated Rac1 activation. We also demonstrated that AKAP12, but not AKAP220, is important for subsequent cAMP-mediated endothelial barrier improvement. Finally, we showed that AKAP220 forms a complex with PKA, ß-catenin and VE-cadherin, and therefore can spatially link cAMP signaling to endothelial adherens junctions.

### cAMP signaling needs to be compartmentalized to exert barrier protective effects

cAMP is well established to be important for barrier formation and maintenance [7], [33]. Furthermore, a variety of barrier destabilizing mediators decrease cAMP levels leading to increased permeability [26], [31], [43]. This decrease in cAMP can be initiated by a rapid Ca^2+^-influx [26], [33], [44] which subsequently may inhibit AC activity [45]. Downstream of cAMP, Rac1 is an important signaling molecule for barrier stabilization [7], [35], [46]. Two distinct signaling pathways involving on one hand PKA and on the other hand Epac1/Rap1 have been shown to merge on and activate Rac1 [47]. Rac1 via a plethora of mechanisms [35], [40], [46] induces cortical actin remodeling and reorganization of intercellular junctions which ultimately affects paracellular permeability [1], [34]. Not surprisingly, it is important where in the cell cAMP is generated in order to provide barrier protective effects [48]. cAMP generated by membrane-attached ACs is protective, whereas cAMP increased by a soluble AC is not. The latter in contrast increases permeability [49]. Thus, to provide distinct effects, cAMP signaling needs to be tightly regulated in space and time. Beside ACs, other key players involved in this regulation are PDEs, which locally hydrolyze cAMP [50]. Similarly, AKAPs facilitate compartmentalization of PKA signaling downstream of cAMP. Our data provide a mechanism, by which the function of PKA can be directed to cell junctions.

### AKAPs are essential for maintenance and stabilization of endothelial barrier properties

Under resting conditions, TAT-Ahx-AKAPis destabilized barrier functions both *in vitro* and *in vivo*. This effect was qualitatively similar in two microvascular cell types and postcapillary venules, indicating that AKAP function is an important factor for endothelial barrier maintenance. Similar to our observation, a recent study demonstrated that low expression of AKAP12 may lead to blood-retinal barrier dysfunction [21]. Further investigations in this direction reported the role of AKAP12 in maintenance of the vascular integrity by modulation of the actin cytoskeleton dynamic through PAK2 and AF6 [20]. Another member of the AKAP-family, i.e. AKAP9 was also discovered to be required for microtubule growth, integrin adhesion at cell-cell borders and endothelial barrier function via Epac1-dependent pathway [51]. Thus, besides PKA, AKAPs can also be associated with Epac1 [51], [52]. Therefore, AKAPs may serve as coordinators not only of PKA- but also of Epac1- induced regulation of endothelial barrier properties [52].

Furthermore, we found that inhibition of AKAP function via TAT-Ahx-AKAPis also interfered with barrier stabilization in response to increased cAMP. In HDMEC, this approach was effective to revert F/R-induced barrier stabilization. In line with that, earlier we reported that incubation with a cell permeable PKA inhibitor blocked the F/R-mediated increase in TER [36]. Herein, we also showed that depletion of AKAP12 but not of AKAP220 significantly decreased cAMP-mediated endothelial barrier integrity as examined by TER (Figure 4). Moreover, simultaneous depletion of AKAP12 and AKAP220 but not of a single AKAP impaired cAMP-mediated Rac1 activation (Figure 6) which is indicative for a redundant function of those AKAPs in the regulation of Rac1 activity. Taken together, these results also demonstrate that AKAP12 may interfere with cAMP-mediated endothelial barrier stabilization in a manner which at least in part is independent of Rac1 (because AKAP12 down- regulation did not alter Rac1 activation). In agreement with this presumption is our recent study revealing that F/R- induced Rac1 activation and barrier augmentation were not affected by the Rac1 inhibitor NSC-23766. Therefore, we argue that GTPases other than Rac1 may also account for the F/R- induced enhancement of endothelial barrier properties [34]. Additionally, one can speculate that besides Rac1, AKAP12 may take part in different cAMP-induced signaling pathways involved in endothelial barrier stabilization. In this respect, a recent study determined AKAP12 molecule as a dynamic platform for signal transduction complexing a number of signaling molecules such as PKA, PKC, calmodulin, F- actin and ß-adrenergic receptors [53].

Similar to AKAP12, we also showed that depletion of AKAP220 impaired the function of the endothelial barrier in MyEnd cells. However, the effect of silencing specific AKAPs was less prominent than the one observed upon TAT-Ahx-AKAPis application (Figure 4). This supports the idea that several AKAPs including AKAP220 and AKAP12 are involved in modulation of endothelial barrier function.

### AKAP220 contributed to endothelial barrier integrity by forming a multivalent complex that links cAMP signaling to adherens junctions

Besides PKA anchoring, several AKAPs were found to act as scaffolding proteins thereby participating in various signal transduction processes. Formation of multivalent complexes provides a high level of specificity and temporal regulation to cAMP/PKA signaling [17]. As mentioned above, we examined the role of AKAP220 which was already reported to organize multivalent complexes. In this respect, AKAP220 was shown to form a complex with IQGAP1 and E-cadherin in MCF-7 cells and to link cAMP signaling to cell adhesion [25]. Furthermore, recent investigations provided evidence that AKAP220 forms a complex with IQGAP2 that favors PKA-dependent recruitment of Rac1 to strengthen cortical actin [24]. Thus, AKAP220 not only provides substrate specificity by tight subcellular localization of PKA, but also regulates and restricts the activity of several effectors which are part of this complex [24], [54], [55], [56]. Similar to AKAP79/150, which was found to localize on the cell membrane and to assemble a ternary complex with E-cadherin and ß-catenin in epithelial cells [57], we detected AKAP220 to co-immunoprecipitate with VE-cadherin and ß-catenin as well as to localize at cell borders similar to VE-cadherin, PKA and Rac1 in microvascular endothelial cells (Figure 2 and 3). Moreover, we demonstrated that F/R- mediated endothelial barrier stabilization was paralleled by increased membrane localization and association of PKA with AKAP220 and VE-cadherin in a complex. The latter observations are consistent with the idea that cAMP via PKA may allow compartmentalized Rac1 activation close to adherens junctions and the cortical actin cytoskeleton. This may be physiologically relevant because TAT-Ahx-AKAPis induced prominent cytoskeletal rearrangement and VE-cadherin interdigitation under conditions of a destabilized endothelial barrier. These effects were associated with decreased PKA, AKAP220, and Rac1 membrane staining, as well as reduced Rac1 activity (Figure 2 and 5). Additionally, TAT-Ahx-AKAPis decreased the association of AKAP220, VE-cadherin and ß-catenin with PKA demonstrating that AKAPs are required to localize PKA to endothelial adherens junctions (Figure 3). Consistent with our assumptions is a study demonstrating that PKA, Epac1, PDE4D and AKAP79 are recruited to VE-cadherin-based complexes in response to cell-cell-contact formation [37].

In conclusion, we showed that AKAPs, and specifically AKAP12 and AKAP220, contribute to regulation of microvascular endothelial barrier function in Rac1- dependent and – independent manner. Our data also indicate that AKAP220 forms a multivalent protein complex linking cAMP signaling to adherens junctions.

## Supporting Information

Figure S1 **TAT-Ahx-AKAPis interfered with endothelial barrier function in MyEnd cells.** (A) shows TER time courses for different conditions applied in MyEnd cell monolayers. In comparison to control and TAT-Ahx-mhK77- treated endothelial cells, monolayers subjected to TAT-Ahx-AKAPis significantly decreased TER over time. The effect was slower than in HDMEC cells but prominent approximately 600 min after initial application. Moreover, in vehicle and TAT-Ahx-mhk77- treated monolayers F/R application led to a progressive TER increase over time. In contrast, TER in TAT-Ahx-AKAPis- pretreated monolayers transiently increased after addition of F/R but declined afterwards to finally reach values similar to control conditions after 450 min. (B) outlines the results after 800 min, the time point at which the monitored effects reached their peaks. Data were collected from more than three independent experiments (N ≥3). * p≤0.05 and *** p≤0.001 indicate statistically significant difference between examined groups; n.s., not significant.
(TIF)
