# Supplementary figures and images for: PKA Compartmentalization via AKAP220 and AKAP12 Contributes to Endothelial Barrier Regulation
Source: PLoS One. 2014 Sep 4;9(9):e106733. doi: 10.1371/journal.pone.0106733 (PMC4154725; doi:10.1371/journal.pone.0106733)

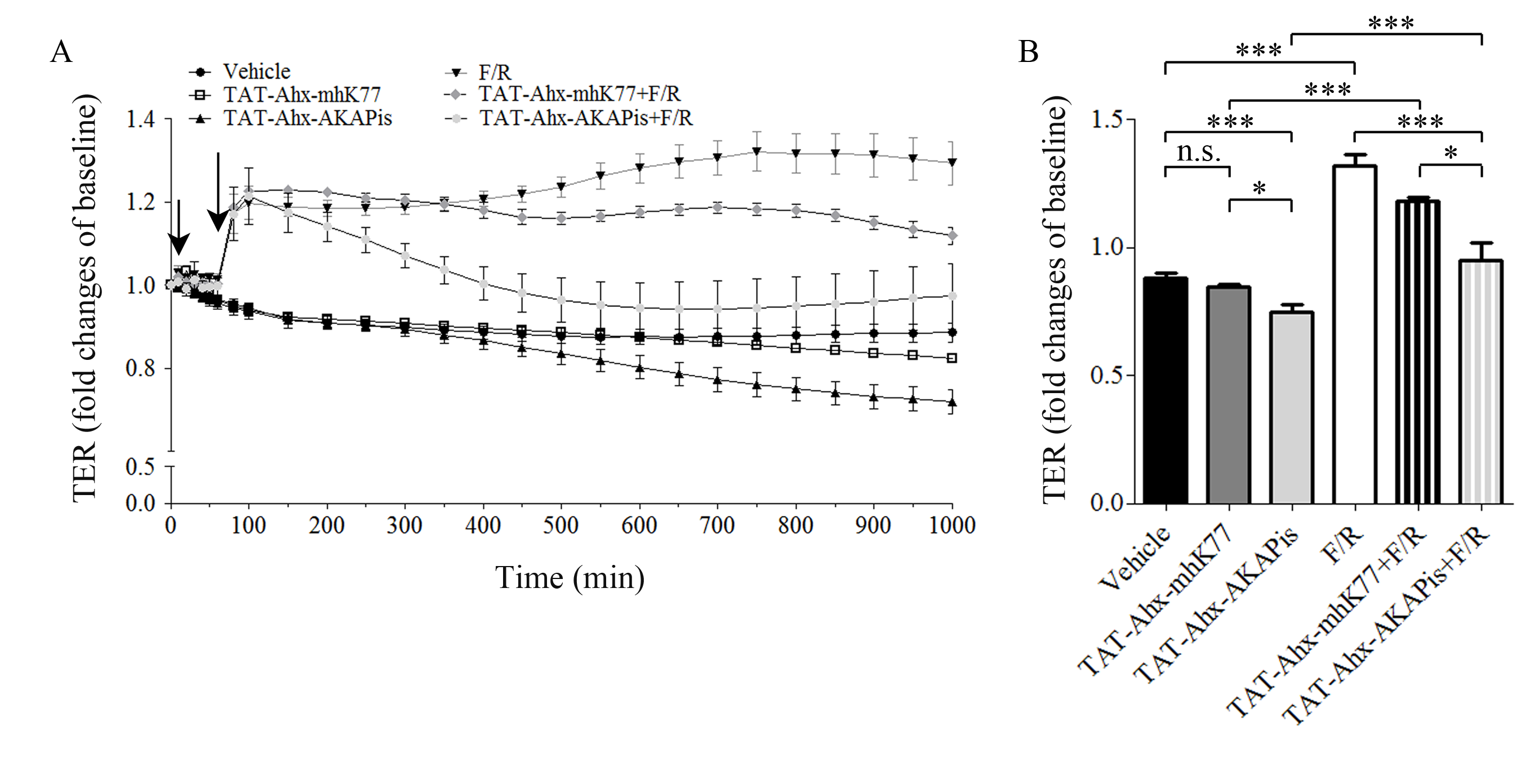

Supplement: Figure S1 — TAT-Ahx-AKAPis interfered with endothelial barrier function in MyEnd cells. (A) shows TER time courses for different conditions applied in MyEnd cell monolayers. In comparison to control and TAT-Ahx-mhK77- treated endothelial cells, monolayers subjected to TAT-Ahx-AKAPis significantly decreased TER over time. The effect was slower than in HDMEC cells but prominent approximately 600 min after initial application. Moreover, in vehicle and TAT-Ahx-mhk77- treated monolayers F/R application led to a progressive TER increase over time. In contrast, TER in TAT-Ahx-AKAPis- pretreated monolayers transiently increased after addition of F/R but declined afterwards to finally reach values similar to control conditions after 450 min. (B) outlines the results after 800 min, the time point at which the monitored effects reached their peaks. Data were collected from more than three independent experiments (N ≥3). * p≤0.05 and *** p≤0.001 indicate statistically significant difference between examined groups; n.s., not significant. (TIF) [file pone.0106733.s001.tif]
